# Efficacy of Chitosan and Chlorhexidine Mouthwash on Dental Plaque and Gingival Inflammation: A Systematic Review

**DOI:** 10.7759/cureus.23318

**Published:** 2022-03-19

**Authors:** Indumathy Pandiyan, Pradeep Kumar Rathinavelu, Meignana I Arumugham, Srisakthi D, Arthi Balasubramaniam

**Affiliations:** 1 Public Health Dentistry, Saveetha Dental College and Hospital, Chennai, IND

**Keywords:** plaque control, periodontitis, chlorhexidine, gingival inflammation, chitosan

## Abstract

Mouthwash is the effective chemical plaque control mechanism being practiced globally. Teeth and tongue discoloration, a temporary change in taste perception, an increase in calculus deposits, a burning sensation, and genotoxicity of buccal epithelial cells are all possible side effects. This review evaluates the efficacy of chitosan mouthwash in comparison to chlorhexidine mouthwash in combating plaque accumulation and gingival inflammation. Electronic databases such as Medline, Cochrane, LILACS, TRIP, Google scholar, and clinical trial registries (CTRI) for ongoing trials were searched with appropriate medical subheadings (MeSH) and search terms. Randomized clinical trials comparing the efficacy of chitosan mouthwash and chlorhexidine mouthwash on dental plaque accumulation and gingivitis were included. The outcome variables of interest were plaque index, gingival index, gingival bleeding index, and colony-forming unit (CFU/ml). All data from the included studies were extracted in a customized extraction sheet. The risk of bias across the studies was assessed using the Cochrane tool for intervention (ROB-2), which consisted of six domains. Of the included three studies, we found one study with an overall low risk of bias and two studies with an overall high risk of bias across the domains. Though there was a significant reduction in plaque accumulation, gingival inflammation, and colony-forming units on the use of chitosan mouthwash and chlorhexidine mouthwash separately, all three included studies reported that a combination of both be more effective.

## Introduction and background

Plaque‐induced gingivitis is a very common periodontal disease that is seen in everyday dental practice. It is caused by the build up of microbial biofilms on the surfaces of teeth, and poor or insufficient oral hygiene is the primary cause [1]. Plaque-induced gingivitis is characterized by redness, puffiness, and a proclivity for easy bleeding when brushing or flossing. If left untreated, gingivitis, the first stage of periodontal disease, would spread and infiltrate the soft and bony supporting components of the teeth, eventually leading to tooth loss. Plaque-induced gingivitis treatment to prevent and reduce plaque accumulation by a number of approaches that enhance oral hygiene [2]. These include tooth brushing, flossing, tooth cleaning sticks, oral irrigators, and/or professional scaling and polishing to mechanically remove dental plaque [3].

However, due to subjective variability, the efficacy of mechanical plaque management remains debatable [4,5]. In such circumstances, antimicrobial mouthwashes should be used in conjunction with mechanical oral hygiene methods [6]. Antimicrobial mouthwashes prevent plaque development by decreasing oral bacteria's growth, metabolism, and colonization [7]. Mouthwashes containing chlorhexidine gluconate are the most commonly used supplement to mechanical intervention in the treatment of gingivitis. It has been shown to be extremely effective in reducing plaque accumulation and is considered the gold standard for plaque control [8].

Long-term usage of chlorhexidine, however, has been linked to a variety of side effects, including tooth and tongue discoloration, a temporary change in taste perception, an increase in calculus deposits, a burning sensation, and genotoxicity of buccal epithelial cells [6,9]. A variety of natural products have been incorporated into dental for plaque control and caries prevention due to increased antibiotic resistance and adverse effects of some antimicrobials on the one hand, and the safety, availability, and relatively low costs of natural products on the other hand [10].

Chitosan is a natural polymer made from the alkaline hydrolysis of chitin, a natural chemical present in exoskeletons of arthropods, crab shells, and insect cuticles. Chitosan and related nanoparticles have gotten a lot of attention in the pharmaceutical, food, agriculture, textile, and tissue engineering industries because of their inherent biocompatibility, biodegradability, and lack of toxicity. Chitosan contains antibacterial, antioxidant, wound healing, and mucoadhesive properties [11,12]. Chitosan has an anti-adherence activity which causes bacterial surface modifications, alterations in bacterial surface ligands expression levels, and gets adsorbed to the hydroxyapatite crystals in the tooth surface. These characteristics are responsible for the bactericidal and bacteriostatic properties of chitosan [13].

The interaction of cationic chitosan with the anionic cell surface, increasing membrane permeability and cellular material leakage from the cell may be the antibacterial mechanism of chitosan. Chitosan may also interfere with the production of mRNA and the embedding of proteins [14]. Due to its outstanding features such as absorbability, malleability, and cohesive threshold concentration to store and gradually release pharmaceuticals with optimal resorption, it has previously been used as a carrier system for the local administration of various drugs [15]. It also has anti-inflammatory properties because it affects prostaglandin E2 levels [16]. Since evidence suggest chitosan to be less cytotoxic and genotoxic this review has been directed to find its efficacy against chlorhexidine [17,18].

The objective of the review is to evaluate the efficacy of chitosan vs chlorhexidine Mouthwash on dental plaque and gingival inflammation.

## Review

All randomized controlled and clinical trials comparing the efficacy of chitosan and chlorhexidine mouthwash were included. Case reports, case series, in-vitro, and animal studies that measured the plaque accumulation and gingivitis were excluded. Periodontally healthy individuals aged 30 years and above of both genders were the populations in each of the included studies. The primary outcomes of the review are plaque index, gingival index. The secondary outcome is the total bacterial count as colony forming units (CFU/mL).

A detailed search strategy for each database to find out studies for this review was developed. Both free-text terms, MeSH terms and a combination of both was used to search in each database. Electronic searches were conducted on Medline, Cochrane, LILACS, TRIP, and Google scholar. No language restrictions were placed. However, studies published from 2011 to 2021 were included. The search strategy of the Medline database is given in Appendix 1. World Health Organization International Clinical Trials Registry Platform was searched to identify for ongoing trials. Also, a hand search was made with the help of a librarian in the following journals: Journal of Periodontology, Journal of Clinical Periodontology, Journal of Periodontal Research.

The authors assessed the obtained studies to find whether they met the selection criteria. All randomized controlled and clinical trials that compared the effectiveness of chitosan and chlorhexidine on plaque accumulation and gingival inflammation which met the selection criteria were included. Case reports, case series, in-vitro studies, and animal trials that measured plaque and gingival index were excluded. Any disagreement between the authors was resolved by discussion. Each author screened for the title and abstract of each article identified through the search strategy. If there were insufficient data in the title and abstract, the full text of the publications was collected in order to make a clear choice.

Each of the review authors independently extracted data with the help of a specially designed data extraction sheet. The following data were recorded for each study: study ID, study design, sample size, participants and group, methodology, parameters, statistical analysis, and results. Any disagreements in the data extraction were sorted out by discussion.

Assessment of methodological quality of included studies was performed. Each author independently assessed the risk of bias for each included study, any disagreement was resolved by discussion to arrive at a consensus. Revised Cochrane Risk of Bias Tool for Randomized Trials (RoB‑2) [19] to assess the risk of bias for included studies. Bias arising from the randomization process, bias due to deviations from the intended interventions, bias due to missing outcome data, bias in the measurement of the outcome, and bias in the selection of the reported results were all domains used to assess the methodological quality of the included studies. The overall score was given for each study based on the scores of each domain. Low RoB for studies for which we identified all domains as being “low risk.” Studies for which we identified one domain with some concerns come under “some concerns.” High RoB for studies for which we identified one or more domains as being “high risk” and more than two domains as “some concerns.”

Since, there was difference in the methodological assessment for plaque index, gingival index and also availability of insufficient data to pool the results, meta-analysis has not been performed. Thus, forest plot for pooled results and funnel plot for publication bias were not performed.

**Figure 1:**
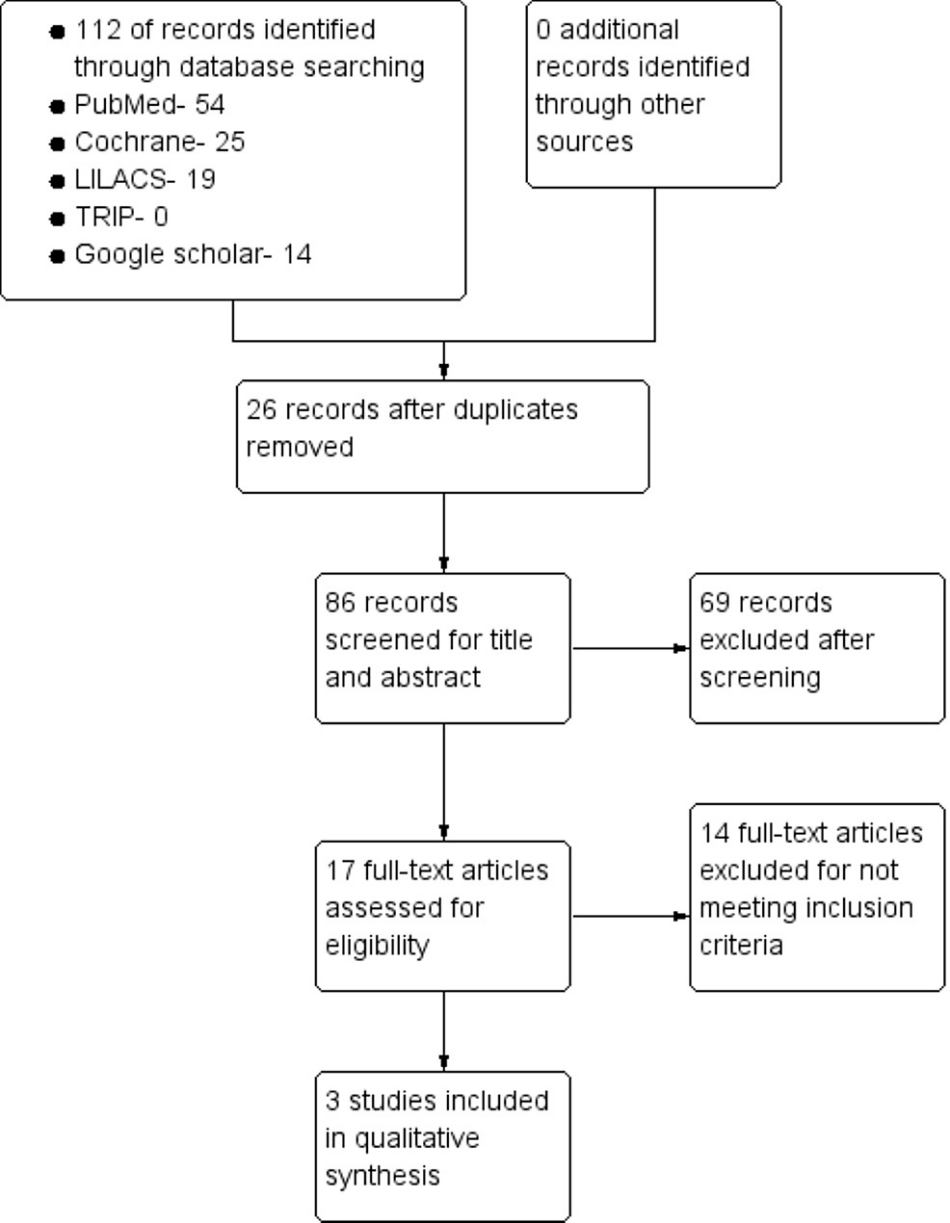
Flowchart of search

A search strategy yielded 112 publications from various databases. On removing 26 duplicate records, we landed up with 86 records. These 86 records were screened for title and abstract, of which 69 records were not suitable for this review. About 17 remaining records, 14 records were excluded for not meeting the inclusion criteria. Thus, three records remained for qualitative analysis in the review as shown in Figure 1.

The authors individually extracted data with the help of a data extraction form and entered all the details into a spreadsheet. The following attributes were recorded for each of the included studies: study ID, study design, sample size, participants and group, methodology, parameters, statistical analysis and results of the plaque index and gingival inflammation. The detailed characteristics of the included studies and the method of assessment and follow‑up data were presented in Table 1. Among the three studies, two studies are Interventional clinical trials and one study was a randomized controlled trial. The parameters analyzed were gingival index, plaque index, bleeding index and colony forming unit.

**Table 1:** Summation table of the included studies Abbreviation: CHX- Chlorhexidine; CHT- Chitosan; CFU- Colony Forming Unit

Study ID	Year	Evaluation period	Study groups	Method of evaluation	Outcome	Limitations/future scope
Vilasan et al. [20]	2020	3 months	1. Group 1, 20 patients rinsing with 20 ml of 0.2% chlorhexidine twice daily for 3 months were allocated. 2. Group 2-10 patients rinsing with 20 ml of 0.5% chitosan twice daily for 3 months were allocated. 3. Group 3- 10 patients rinsing with 10 ml of chlorhexidine chitosan combination twice daily for 3 months were allocated	1. Plaque index using Turesky Gilmore Glickman modification (1970) of the Quigley and Hein (1962) index. 2. Gingival index using Loe and Silness index (Loe and Schiott, 1970) 3. Bleeding on probing using Ainamo and Bay index.	The combination of chitosan and chlorhexidine showed a statistically significant reduction (p<0.05) in plaque indices from baseline at all-time intervals when compared to that of chlorhexidine or chitosan alone.	The study was carried out on a small sample size with short evaluation time. It did not have any microbiological analysis and toxicity testing. No attempt was made to find out the exact mechanism of chlorhexidine and chitosan combination.
Mhaske et al. [21]	2018	4 days	1. Group I included 15 subjects who used 0.2% CHX 2. Group II included 15 subjects who used 2% CHT solution 3. Group III involves 15 subjects who used 0.2% CHX/2% CHT combination.	Plaque index, Gingival index and *Streptococcus mutans* count	1. Plaque index was lowest in group I at day 0, while it was highest in group III. 2. At day 4, PI was highest in group II, while lowest in group III. 3. Gingival index was lowest in group I and highest in group II at day 0, and lowest in group I and highest in group III at day 4. 4. Both chitosan and chlorhexidine were found to be effective in controlling plaque. However, a combination of both provides even better results.	Less sample size and low follow up days
Nair et al. [22]	2017	7 Days	1. CHX group A: 30 seconds mouth rinsing with 15 ml of CHX mouthwash for 20 patients. 2. CHT group B: 30 seconds mouth rinsing with 20 ml of CHT in 10 ml of water for 20 patients.	Colony forming unit (CFU)	The mean CFU count reduction after using 0.12% CHX and 2% CHT for one week were 3.563X102 and 3.714X102 respectively. Both the mouthwashes were effective in reducing the total bacterial count after one week.	Less follow up days. Specific microorganism name was not mentioned in the study.

**Figure 2:**
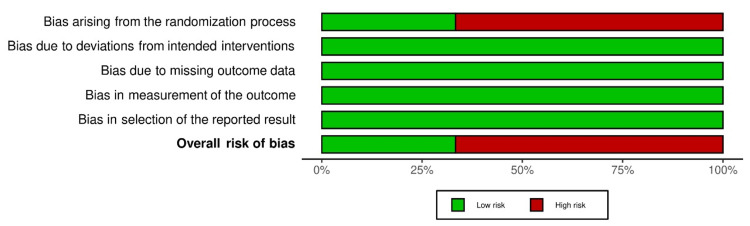
Risk of bias - 2 (ROB-2) presented as percentages across all included studies

**Figure 3:**
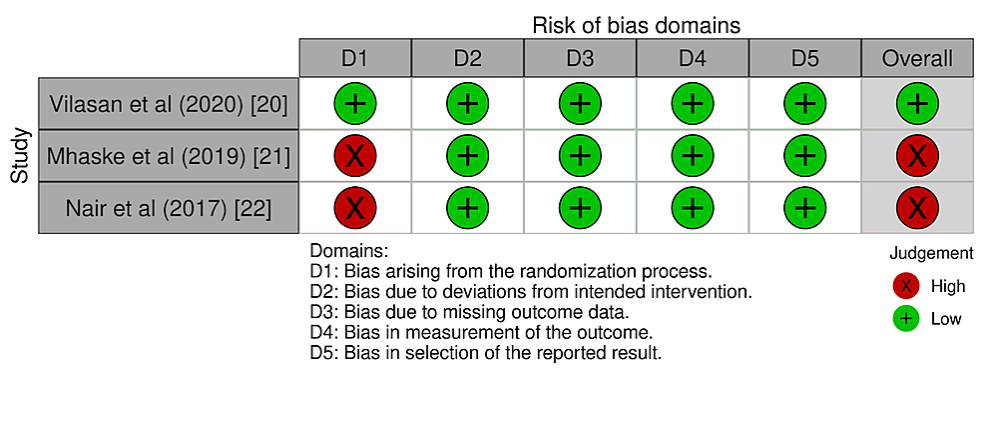
Risk of bias - 2 (ROB-2) for each included study

In Figures 2, 3, the review authors' judgments on each RoB-2 were shown as percentages across all included research, and the RoB-2 for each included study was presented as a summary. Among the three included studies, one study had low risk of bias [20] and two other studies has high over all risk of bias [21,22].

Summary of the main results

To the best of our knowledge, this is the first systematic review to compare the efficacy of chitosan mouth rinse versus chlorhexidine in preventing plaque and gingivitis. Overall, chitosan is helpful in reducing plaque and gingival irritation, according to the findings of this systematic review of 145 samples. The quality of the included studies, as well as the high heterogeneity among them, must be considered when weighing the results of this review. The reduction of plaque scores and gingival inflammation were the major endpoints of this study.

Despite the fact that most individuals practice brushing their teeth at least twice a day, the prevalence of gingivitis and chronic periodontitis remains high in most populations around the world [23]. Effective plaque control is well acknowledged as a critical aspect in the prevention and treatment of periodontal disorders [2,7]. Despite the fact that mechanical oral hygiene is the simplest and most effective technique for plaque reduction, the majority of adults do not brush or floss their teeth effectively [4,5]. Mouth rinses have been shown to be efficient in blocking and lowering gingival plaque formation when used in conjunction with mechanical oral hygiene [7].

In the current review, two studies [19,20] found that a combination of chlorhexidine mouthwash and chitosan mouthwash was effective in reducing plaque and gingival score, whereas Nair et al. [22] evaluated the in vivo effect of CH and chitosan on plaque microbial and found a mean colony-forming unit count reduction after using 0.125% CHX and 2% chitosan for one week and concluded that both are effective and Van Strydonck et al. [24] compared 0.12% CHX to 0.05% cetylpyridinium chloride and 0.2% CHX after three days and found no significant difference in plaque accumulation in either group. Costa et al. [25] stated that chitosan is efficient against the majority of bacteria and recommended it as a replacement for standard mouthwashes.

Decker et al. [26] investigated the effects of CHX on plaque combinations in order to develop antiplaque techniques. In that investigation, CHX (0.1%) was utilized as a positive control, saline was used as a negative control, and two CHT derivatives were linked to Streptococci sanguis for two minutes with their CHX combination. According to their findings, the CHX & CHT combination was more effective than CHX alone because it combined the bioadhesive qualities of CHT with the antibacterial activity of CHX, resulting in a synergistic antiplaque effect that was superior to CHX alone. The antiplaque action of chitosan, according to Decker et al. [27] and Costa et al. [28], is due to its antiadhesive property toward microbes. Chitosan, according to some researchers, can be used efficiently in dentifrices to promote oral hygiene since it decreases plaque by 70% [29].

In a randomized clinical experiment, Uraz et al. [30] investigated the clinical and microbiological effects of chitosan on dental plaque and discovered a reduction in microbiological count (*S. mutans* and *C. albicans* levels) in both the CH and chitosan groups. Chen and Chung [31] tested the bactericidal activity of chitosan in vivo and in vitro at various temperatures (25-37°C) and pH levels (pH 5-8). They discovered that chitosan has antibacterial properties equivalent to commercial mouthwashes. They concluded that in the future, water-soluble chitosan could be a viable alternative to commercial mouthwashes.

Costa et al. [28] investigated the possible use of high- and low-molecular-weight chitosan as an oral antibacterial agent and found that efficiency decreased only little after a week. They also discovered that chitosan could block the formation of biofilms by two microorganisms and could act on mature biofilms, resulting in a 94% reduction in biofilm survival. Giunchedi et al. [32] looked examined CHX buccal tablets made from drug-loaded CH microspheres. Combining CHT microspheres with CHX as a controlled drug delivery system not only extended the drug's release in the oral cavity, but also increased CHX's antibacterial effectiveness.

Strengths and weaknesses of the review

This review included all randomized controlled and clinical trials and excluded case reports, case series, in-vitro, animal studies and ex-vivo studies. Because this evaluation looked at all human in-vivo trials, it has a lot of therapeutic application. Every precaution was taken to reduce bias at every stage of the evaluation. To discover all relevant studies, we searched electronic databases and trial registries with no language constraints. We used the Cochrane Risk of Bias - 2 tool to assess the methodological quality of the included studies, which has five categories and one overarching area that offers exploratory information on the risk of bias. One included study had low risk of bias and two included studies has high risk of bias. The primary outcomes of the review are plaque index and gingival index. Though each study evaluated plaque index and gingival index as scores, the criteria of the indices used are different. Therefore, no quantitative analysis and data synthesis, investigation of heterogeneity, sensitivity analyses were performed. Also, the present review failed to search for other databases such as Excerpta Medica database (EMBASE) and EBSCO. All included studies are from one country (India), the results of the review may or may not be generalizable to other countries. Thus, the applicability of the results of this review is possible to the Indian population.

## Conclusions

Based on the present review, both chitosan and chlorhexidine are found to be effective in controlling plaque and gingival inflammation. However, a combination of both provides even better results. Chitosan can be used as an alternative mouthwash. Further, randomized controlled trials following the CONSORT guidelines from a different population with different cultural and racial variations are needed to validate the effectiveness of chitosan on plaque accumulation and gingival inflammation.

## Appendices

**Table 2:** Search Strategy (Medline)

S.No	Query	Results
1	((((((((((("gingivitis*"[Title/Abstract]) OR ("Gingival hemorrhage*"[Title/Abstract])) OR ("Dental plaque"[Title/Abstract])) OR ("gingivit*"[Title/Abstract])) OR (plaque[Title/Abstract])) OR ("biofilm"[Title/Abstract])) OR ("microorganism"[Title/Abstract])) OR ("microflora"[Title/Abstract])) OR ("gingival pocket"[Title/Abstract])) OR ("gingival pockets"[Title/Abstract])) OR ("pseudopocket"[Title/Abstract])) OR ("gingiv*"[Title/Abstract])	215,610
2	(("gingivitis"[MeSH Terms]) OR (gingival hemorrhage[MeSH Terms])) OR (dental plaque[MeSH Terms])	26,790
3	((chitosan[Title/Abstract]) OR ("chitosan mouthwash"[Title/Abstract])) OR (chitosan mouth rinse[Title/Abstract])	30,678
4	chitosan[MeSH Terms]	20,820
5	((((("chlorhexidine mouthwash"[Title/Abstract]) OR ("chlorhexidine mouthwashes"[Title/Abstract])) OR ("chlorhexidine mouth wash"[Title/Abstract])) OR ("chlorhexidine mouth washes"[Title/Abstract])) OR ("chlorhexidine mouthrinse"[Title/Abstract])) OR ("chlorhexidine mouthrinses"[Title/Abstract])	491
6	"chlorhexidine"[MeSH Terms]	8,492
7	(((((("plaque index"[Title/Abstract]) OR ("plaque index score"[Title/Abstract])) OR ("plaque index of sillness and loe"[Title/Abstract])) OR ("plaque index scores"[Title/Abstract])) OR ("gingival index"[Title/Abstract])) OR ("gingival index score"[Title/Abstract])) OR (gingival index scores[Title/Abstract])	6,582
8	(((dental plaque index[MeSH Terms]) OR (dental plaque indexes[MeSH Terms])) OR (gingival index[MeSH Terms])) OR (gingival indexes[MeSH Terms])	11,223
9	(#1) OR (#2)	223,330
10	(#3) OR (#4)	31,418
11	(#5) OR (#6)	8,665
12	(#7) OR (#8)	14,231
13	(((#9) AND (#10)) AND (#11)) AND (#12)	3
